# RACIAL-ETHNIC DIFFERENCES IN THE EFFECTS OF POSITIVE AND NEGATIVE AFFECT, AND DEPRESSION ON COGNITIVE TRAJECTORIES

**DOI:** 10.1093/geroni/igz038.2419

**Published:** 2019-11-08

**Authors:** Soohyun Park, Su Hyun Shin, Rebecca S Allen, Giyeon Kim

**Affiliations:** 1 The University of Alabama, Tuscaloosa, Alabama, United States; 2 Chung-Ang University, Seoul, Korea, Republic of

## Abstract

Purpose of study: This study investigated (1) whether positive affect(PA), negative affect(NA), and depression are related to trajectories of cognitive functioning among older adults, (2) whether PA or NA could moderate the relationship between depression and cognitive trajectories, and (3) whether there are racial/ethnic differences in the relationships among PA, NA, depression and cognitive trajectories. Design and Methods: Growth-curve modeling was conducted using the sample of 10,289 individuals in the U.S. aged 50 or older from the 2006-2014 Health and Retirement Study. Racial/ethnic groups in this study were non-Hispanic Whites (NHW, n=8.009), African Americans (AA, n=1,428), Hispanics (n=611), and others (n=241). Results: After adjusting for covariates, PA showed positive effect, and depression had negative effect on cognitive functioning trajectories (p < .05, z = 8.76, 95% CI= 0.27, 0.43; p < .05, z = -6.38, 95% CI= -0.19, -0.10). Racial/ethnic minorities (i.e., AA, Hispanics, others) exhibited lower cognitive functioning over time compared to NHW. PA significantly moderated the effect of depression on cognitive trajectories (p < .05, z = - 8.04, 95% CI = -0.19, -0.11), and the protective effect of PA against cognitive decline was pronounced for AA (p < .05, z = 2.75, 95% CI = 0.10, 0.63). Conclusion: Findings suggest that PA may protect against cognitive decline in older adults, providing a buffer against the negative effect of depression or racial/ethnic minority status on cognitive trajectories. Potential intervention strategies are discussed to assist older adults in maintaining and improving PA to promote cognitive health.

